# Complementary assessment of nano‐packaged garlic properties by electronic nose

**DOI:** 10.1002/fsn3.4158

**Published:** 2024-04-09

**Authors:** Alireza Makarichian, Ebrahim Ahmadi, Reza Amiri Chayjan, Doostmorad Zafari

**Affiliations:** ^1^ Department of Biosystems Engineering, Faculty of Agriculture Bu‐Ali Sina University Hamadan Iran; ^2^ Department of Plant Protection, Faculty of Agriculture Bu‐Ali Sina University Hamadan Iran

**Keywords:** aroma, fung, infection, packaging, PCA, storage

## Abstract

It is crucial to initiate appropriate storage conditions for garlic depending on its properties. Fungal contamination can reduce the quality of garlic through changes in its properties which result in its aroma alteration. This study aimed to evaluate the effects of treatments such as fungal infection (FI), material of packaging (MP), and storage duration (SD) on various characteristics of garlic. An electronic nose was used complementarily to trace the aroma changes as a non‐destructive indicator. The *Fusarium oxysporum* (FS), *Alternaria embellisia* (AL), and *Botrytis allii* (BT) fungi were adopted for inoculation. The low‐density polyethylene (LDPE) and silicone nano‐emulsions (SNE) were used for packing samples. The data were analyzed by diverse approaches such as ANOVA, PLS, PCA, LDA, and BPNN. The results revealed that the evaluated properties changed during the storage. The implementation of treatments altered the intensity of these changes. The highest values of weight loss (21.14%), color changes (50.21), and acidity (7.48) were observed in the FS‐infected samples kept in LDPE for 28 days. The accuracy of PCA, LDA, and BPNN in the multivariate analysis of aroma had an increasing‐decreasing trend. The best accuracy of PCA in categorizing the FI and MP treatments achieved in the twelfth day of storage (96%). The optimal accuracy of classifications based on FI and MP treatments was obtained at d#12 (100%) and d#24 (100%), respectively. The PLS exposed that the aroma changes in garlic had a high correlation with the changes of studied properties (*R* ^2^ ≥ .7), except for the mechanical properties.

## Graphical abstract

Garlic inoculated into three fungi and stored in two types of packaging. The aromas' profile of each treatment composition was detected by E‐Nose. The changes in physical, chemical, and mechanical traits were inspected. PCA, LDA, and BPNN were implemented to analyze volatile fingerprints of garlics. PLS was used to correlate between aroma and qualitative traits.

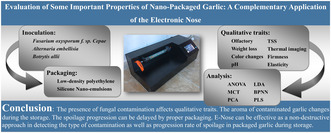

## 1 INTRODUCTION

Highly valued all over the centuries, garlic is one of the most important cultivated spice crops which is an excellent source of various nutrients needed for human health. To enhance the quality of garlic as well as the marketing of that, storage is a prominent element. The storage encourages foodstuffs to preserve their qualitative properties. Besides, the storage helps make economic savings by staving off spoilage as well as foodborne illnesses caused by harmful pathogens. Hence, there is an urge to initiate appropriate storage conditions depending on the different properties of garlic, in such a way that the storage prevents any interventions under optimal conditions of sensory, physical, physicochemical, and health properties.

Petropoulos et al. (2017), defined well all factors that distress the storage of garlic which are related to field and postharvest treatments. One of the most important factors that can affect the quality of garlic is fungal contamination which could lead to the deterioration of clove tissues. This incident becomes more serious during garlic storage. The fungi such as *Alternaria porri*, *Aspergillus niger*, *Botrytis allii*, *Colletotrichum circinans*, *Fusarium oxysporum f*. sp. *Cepae*, *Penicillium* spp., and *Sclerotium cepivorum* are the most common and important plant pathogens which lead to considerable threats to health, quality losses, efficiency reductions, and massive economic damages (Muhammad et al., 2019).

The early identification of fungal contamination in the storage is one of the most critical issues that can prevent a lot of losses. The deterioration associated with fungal contamination can posturize itself in the form of negative effects on the physical, thermal, chemical, and mechanical characteristics. Therefore, the changes in each of these mentioned traits can be employed as an index for the existence of fungal contamination in garlic, which is mainly achieved through destructive experiments.

For crops such as garlic, which has numerous utilizations as a spice, the slightest loss of quality and texture damage causes a change in its aroma. Therefore, the changes in garlic aroma during storage can be used as a non‐destructive indicator to investigate the presence of fungal contamination of the product (Wilson, 2018). Nowadays, the investigation of volatile organic compounds (VOCs) has been widely welcomed in the diagnosis of pathogenic contamination by conventional approaches such as gas chromatography–mass spectrometry (Gao et al., 2022), High‐performance liquid chromatography (Kašpar & Česla, 2022), etc. The conventional techniques are expensive, time intensive, require well‐trained labor, and cannot be suited for online utilization.

Unlike conventional methods, the E‐Nose as a cutting‐edge approach offers an alternative method for the recognition of volatile patterns by emulating the olfaction system of humans (Moshayedi et al., 2023). The E‐Nose draws up several advantages like ease of utilization, high sensitivity, uncostly, and quickness. The performance of conventional techniques is based on the measurement of specific volatiles. In contrast, E‐Nose is mapped out with several non‐selective sensors that link to odor molecules (Xing et al., 2023). The result of such connections is a diverse signal's classes which are sent to a computer to recognize their patterns by multivariate statistics (Aghili et al., 2023). A broad list of E‐Nose applications can be found in the agricultural industry (Seesaard et al., 2022), environmental monitoring (Kumar et al., 2023), food analysis (Oates et al., 2022), and quality control (Pulluri & Kumar, 2022).

To the best of our knowledge, several studies have been conducted to study the effects of treatments such as FI, MP, and SD individually on the qualitative characteristics of the product (Berhe et al., 2023; Etxabide et al., 2021; Tiwari et al., 2021). Besides, several studies have been conducted to study the effect of fungal contamination by the E‐Nose (Astuti et al., 2023; Sun & Zheng, 2023; Wang et al., 2023). Therefore, the principal point of view in this study was to evaluate the simultaneous effect of these treatments on the dominant traits of stored garlic such as physical, chemical, thermal, and mechanical properties. As an innovation, the E‐Nose was used as a novel supervisory approach to monitoring the qualitative characteristics of garlic.

## 2 MATERIALS AND METHODS

### 2.1 Sample provision

The experimental samples were provided directly from farmland. The homogeneous garlic cloves were adopted to be uninvolved of any stain, decay, or damage. The processes related to inoculation were carried out in a bacteria‐free laboratory in the plant pathology laboratory, Department of Plant Protection, Faculty of Agriculture, Bu‐Ali Sina University, Hamedan, Iran. In the sterilization process, the garlic cloves were dipped in 75% ethanol for 30 s and rinsed with sterilized distilled water. To inoculate cloves, the *Fusarium oxysporum f*. sp. *Cepae* (FS), *Alternaria embellisia* (AL), and *Botrytis allii* (BT) fungi were engaged. These fungi were spread on potato dextrose agar at 22°C, 80% relative humidity (RH), and incubated for 168 hours before the inoculation (Li et al., 2011). Garlic cloves were immersed for 30 s in spore suspensions of 1 × 10^6^ spores mL^−1^ related to each fungal pathogen (Fuentes et al., 2013; PalmEro et al., 2012). The non‐infected or control samples (CT) just immersed in sterilized distilled water for 30 s.

Subsequently, to investigate the effect of packaging material on the control of storage conditions, two materials were used for packing samples, that is, LDPE and SNE. The packaged samples had 10 garlic weighing nearly 50 ± 5 g. They were kept at ambient conditions of 22 ± 1°C and 30%–45% RH for 28 days in the rheological laboratory of the Department of Biosystems Engineering, Faculty of Agriculture, Bu‐Ali Sina University, Hamedan, Iran. The treatment combinations were monitored and examined on Days 0 (d#0), 4 (d#4), 8 (d#8), 12 (d#12), 16 (d#16), 20 (d#20), 24 (d#24), and 28 (d#28).

### 2.2 Physical properties investigation

#### 2.2.1 Weight loss (WL)

To calculate the WL during the storage period, three samples of each treatment combination were used separately in the same conditions as the other. The weight of the samples at d#0 was considered as the initial weight (W_0_). Then, every 4 days, the weight of the samples (W_1_) was measured by an analytical balance (mod: GF‐600, ±0.001 g, max 610 g, AND, Japan), and the WL results were calculated by Equation 1.

(1) $$\mathrm{WL}=\left[\left(W_{0}–W_{1}/W_{0}\right)\right]\times 100$$

#### 2.2.2 Color changes (CC)

The CC in garlic cloves was monitored by applying a digital portable colorimeter (hp‐200, Shenzhen Handsome Technology Co., Ltd., China). Three samples of each treatment combination were used separately in the same storage conditions. To diminish the environmental effects on the CC changes, the calorimetric tests were performed at the same time and location circumstances. Eventually, the CC was calculated based on the CIE L*a*b* coordinates and by Equation 2 (Gholami et al., 2017).

(2) $$∆E=\sqrt{∆L^{*2}+∆a^{*2}+∆b^{*2}}$$

### 2.3 Chemical properties investigation

#### 2.3.1 Acidity (pH)

To determine the pH, the whole garlic cloves related to each treatment combination were grated. Because the extract contained a high amount of water, distilled water was no longer added. The extract was then ground to completely homogenize the extract. A 40 μm filter paper was used to filter the collected extract. A pH meter (pHS‐BW, Bante, Italy) was used to measure the pH with a resolution of 0.01 in three replications.

#### 2.3.2 Total soluble solids (TSS)

The TSS was quantified at the ambient temperature of 28 ± 1°C using a refractometer (PAL‐1, Tokyo, Japan) with a resolution of 0.01 and in three replications. Hence, the extract of garlic cloves was placed on the prism, and the TSS results were recorded in % (Gholami et al., 2020).

### 2.4 Thermal properties investigation

The tissue temperature (TT) of cloves during storage was captured by a thermal camera (E40, FLIR, Italy). The camera features a resolution of 120 × 160 pixels; a frame rate of 60 Hz; a thermal sensitivity of <0.07°C at 30°C; a field of view of 19° × 25°; and a spectral range of 7.5–13 μm. To prevent the adverse effects of environmental conditions, imaging was performed in six replications at a certain time of the day and the same setup elements were utilized in each experiment.

### 2.5 Mechanical properties investigation

The puncture tests were carried out on cloves utilizing a deformation analysis dynamometer (Bbt1‐Fro.5th.D14, Zwick/Roell, Ulm, Germany). The puncture probe penetrated to a certain depth (10% of the sample diameter) into the samples at a speed of 20 mm min^−1^. The firmness (*F*_max_) was considered the first maximum peak of the resulting force‐time diagram. Consistent with Mohsenin (1984), Young's modulus (*E*_mos_) was obtained according to chord modulus so that the slope of the line between the two points was associated with the strain of 10% and 40% on the stress–strain curve (in the elastic range).

### 2.6 Olfactory experiments

**FIGURE 1:**
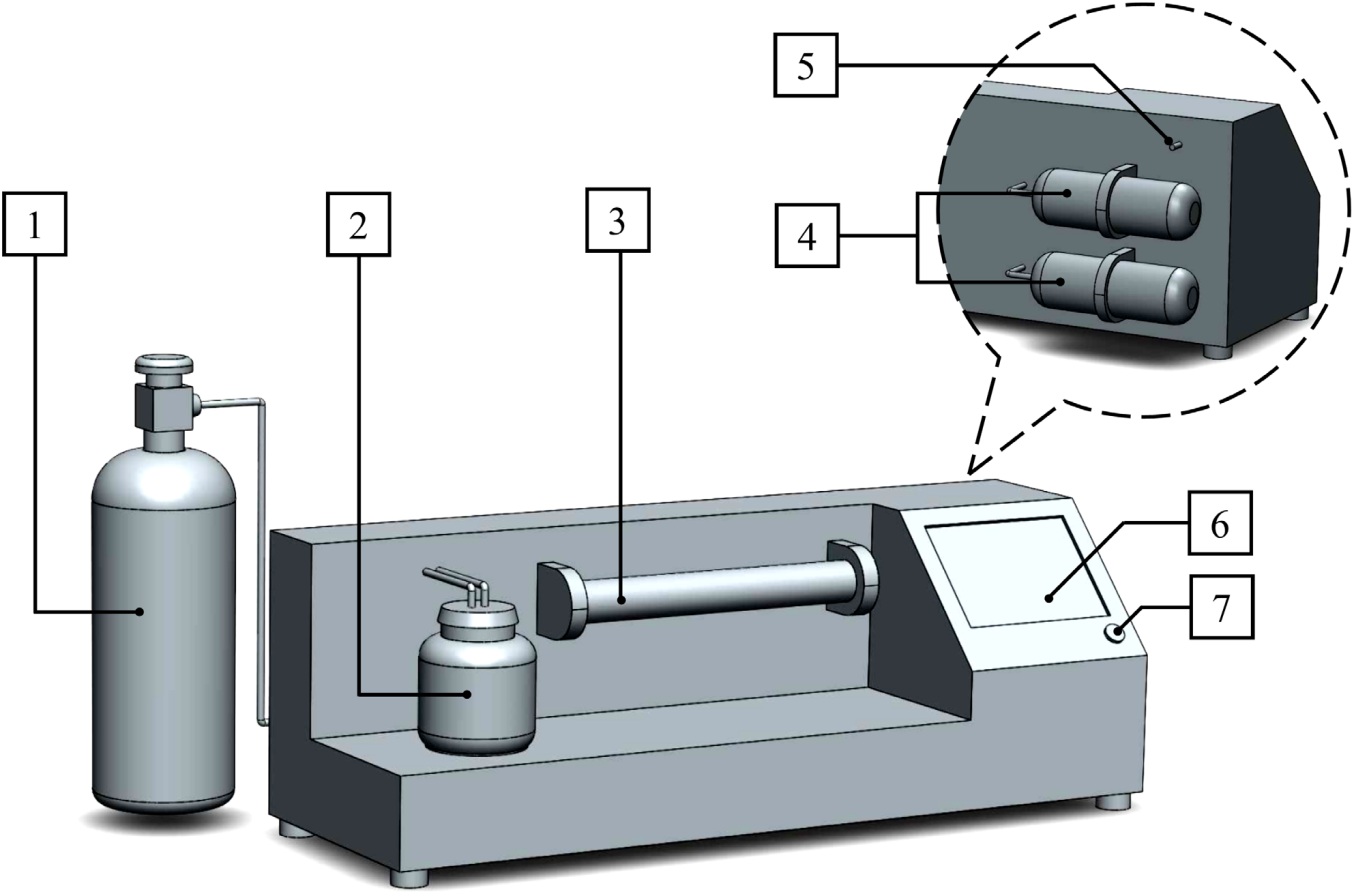
Constructed E‐Nose: (1) oxygen gas tank, (2) sample container, (3) sensor chamber, (4) filters, (5) pump output, (6) touch screen, (7) Power button.

The structural details of the employed E‐Nose (Figure 1) in the olfactory tests have been fully clarified in previous research (Makarichian et al., 2021). Each cycle of the olfactory experiment included the three principal phases of baseline adjustment, headspace insertion, and sensor restoration. The baseline adjustment phase was performed to eliminate any initial errors in the array so that olfactory experiments were started with the least disorders. In the headspace insertion phase, the studied aroma was injected into the sensor chamber. The chemical reactions of the sensor surface with the components of the aroma resulted in sensor responses in the form of voltage changes. The headspace insertion phase was running until the response of the sensors remained unchanged. In the sensor restoration phase, the aroma is discharged from the array headspace to return the sensor response to the baseline. The duration of the baseline adjustment, headspace insertion, and sensor restoration phases were selected experimentally at 40, 60, and 40 s, respectively. The olfactory tests of each sample were carried out in the ambient conditions of 28 ± 1°C and 35%–45% RH.

### 2.7 Data analysis

The influence of three studied treatments, that is, FI, MP, and SD on the different qualitative properties of stored garlic was examined. Statistical analysis was carried out by analysis of variance (ANOVA) test as a factorial test in a completely randomized design. Additionally, the comparison of the means was done by using the Duncan method at the 1% probability value by SAS v9.4 (SAS Inst. Inc., Cary, NC). To model and analyze the correlation between the aroma profiles and evaluated properties, the partial least squares (PLS) was applied. In this approach, the focus was on the relationship between dependent variables (array responses) and independent variables (qualitative traits). Meanwhile, the E‐Nose data were multivariate analyzed and categorized by diverse approaches such as principal component analysis (PCA), linear discriminant analysis (LDA), and back propagation neural network (BPNN). The PCA facilitates extracting useful information from large datasets through dimensionality reduction. The LDA transforms linearly samples belonging to the same category closer while it excludes samples related to different categories. The BPNN is one of the principal neural network algorithms for classification, which assumes the rules noted by the human brain to decode the issues. The unscrambler X 10.4 (CAMO ASA, Norway) software was used to perform the PLS, PCA, and LDA approaches, while the MATLAB R2015b (The Mathworks Inc., Natick, MA) software was run to implement the BPNN.

## 3 RESULTS AND DISCUSSIONS

### 3.1 Changes in physical properties

#### 3.1.1 WL

**FIGURE 2:**
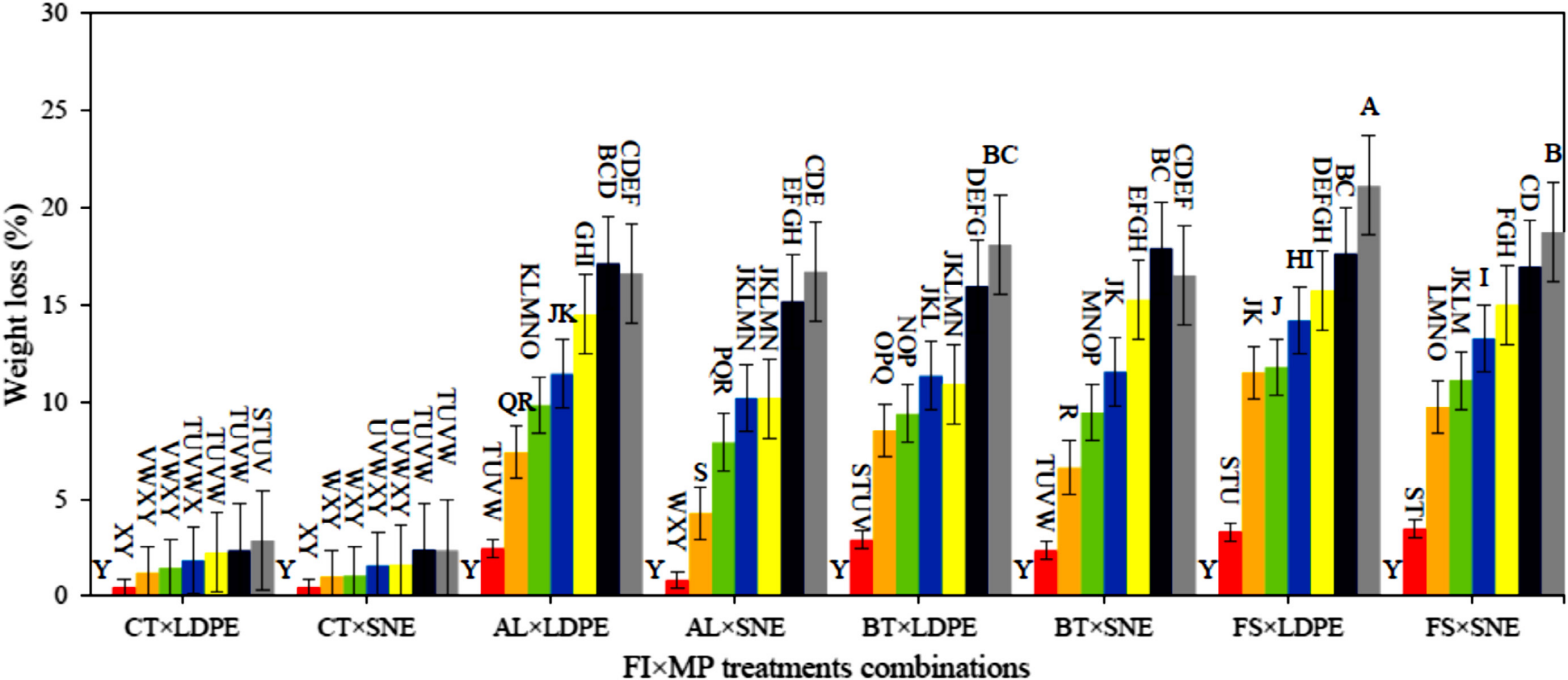
Results of WL alteration of different combinations of FI × MP treatments during storage; ■ d#0, ■ d#4, ■ d#8, ■ d#12, ■ d#16, ■ d#20, ■ d#24, and ■ d#28.

The WL of samples increased over the storage period (Figure 2). The presence of fungal infection caused more WL. The highest WL was related to FS‐infected samples, while the lowest WL was observed in CT ones. The use of different packaging caused significant changes in WL, so the SNE resulted in less WL. The reason for this was changes in respiration conditions caused by different levels of MP treatment. The ANOVA revealed that except for the interaction of MP × SD treatments, all effects of the studied treatments were significant at the level of 1% while the mentioned interaction was significant at the level of 5% (Table 1). The comparison of the means demonstrated a significant difference between the diverse levels of all treatments. The lowest and highest WL was observed in treatment combinations of “CT × SNE × d#4” (0.45%) and “FS × LDPE×d#28” (21.14%), respectively. Lan et al. (2019), stated that the storage time and the use of Nano‐packaging affect the WL of samples during storage, and more WL lies in a longer storage period. In contrast, Nano‐packaging cut off the WL due to its better inhibitory properties.

**TABLE 1:** ANOVA of the treatments on the studied traits of garlic cloves.

Sources	Df	WL	CC	pH	TSS	TT	*F*_max_	*E*_mod_
a	3	2860.92**	5109.48**	1.47**	142.70**	146.45**	987.72**	0.20**
b	1	19.84**	167.87**	0.46**	0.05**	4.29^ns^	197.97**	0.00^ns^
c	7	4231.86**	10940.99**	27.50**	446.08**	1730.27**	4651.58**	0.75**
a × b	3	28.82**	81.01**	0.43**	10.39**	95.35**	176.37^ns^	0.02*
a × c	21	1000.53**	5275.18**	3.06**	146.42**	121.16**	429.35^ns^	0.05^ns^
b × c	7	13.39*	250.36**	1.01**	2.34**	39.35**	176.31^ns^	0.03*
a × b × c	21	60.26**	168.73**	0.81**	10.61**	80.35*	262.73^ns^	0.04^ns^
Error	126	103.20	1.15	1.01	0.71	651.15	11427.92	1.08

Abbreviations: a, Fungal infection; b, packaging material; c, storage stages; ns, no significant difference.

* A significant difference in the level of 5%.

** A significant difference in the level of 1%.

#### 3.1.2 CC

**FIGURE 3:**
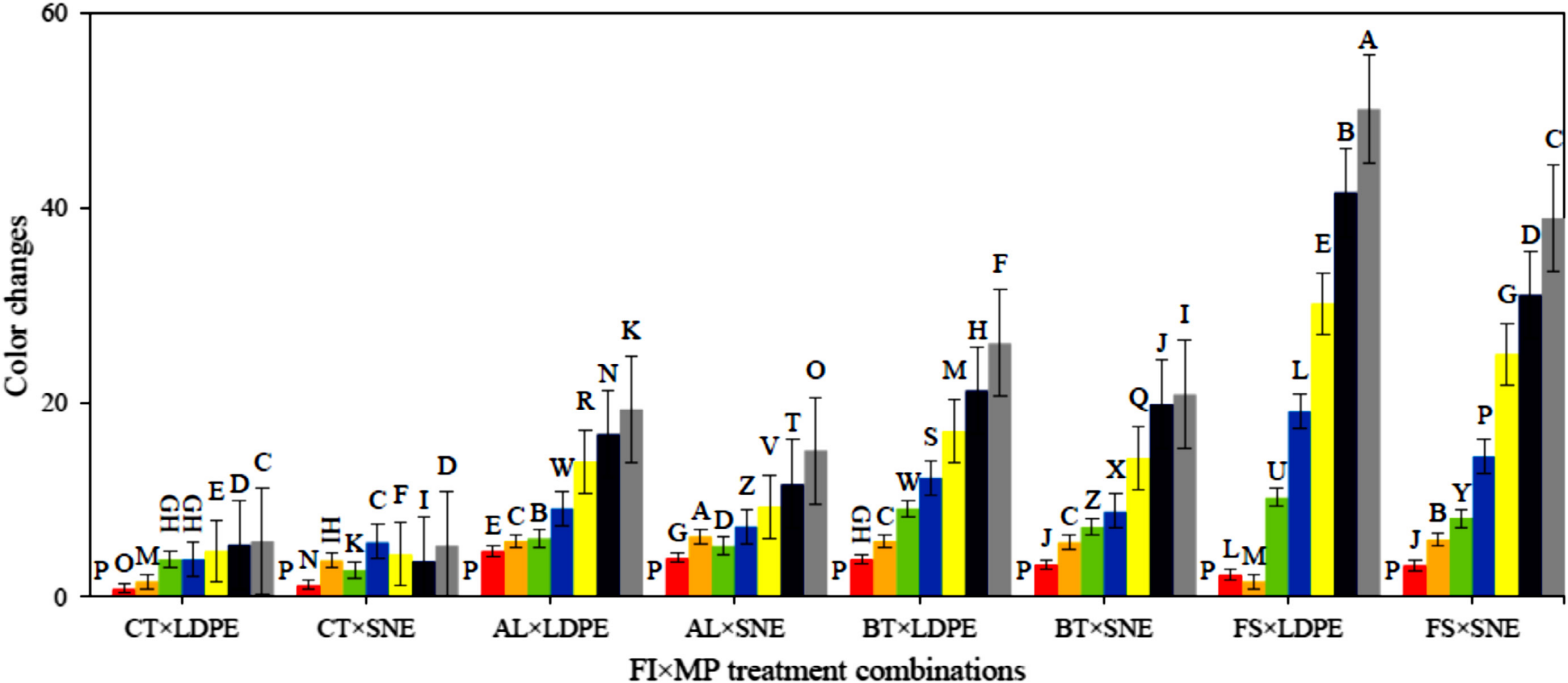
Results of CC alteration of different combinations of FI × MP treatments during storage; ■ d#0, ■ d#4, ■ d#8, ■ d#12, ■ d#16, ■ d#20, ■ d#24, and ■ d#28.

Samples experienced more CC over the storage period (Figure 3). Although the trend of CC in the CT samples was not regular, the CC occurred obviously over the period. The presence of fungal infection caused more CC in samples as much as possible. The use of SNE packaging also caused less CC due to more inhibitory properties than LDPE. The reason could be less respiration in SNE which controlled fungal growth as well as changes in physical condition. The ANOVA disclosed that all effects of the studied treatments on the CC changes were significant (Table 1). The comparison of the means showed that the lowest and highest amount of CC belonged to the treatment combinations of “CT × SNE × d#4” (0.85) and “FS × LDPE×d#28” (50.21), respectively. Numerous researchers have stated that the absence of any dangerous pathogens as well as the use of nano‐packaging increases the quality indicators and retains the color of the product in the storage (Alden et al., 2019; Gómez et al., 2020).

### 3.2 Changes in chemical properties

#### 3.2.1 pH

**FIGURE 4:**
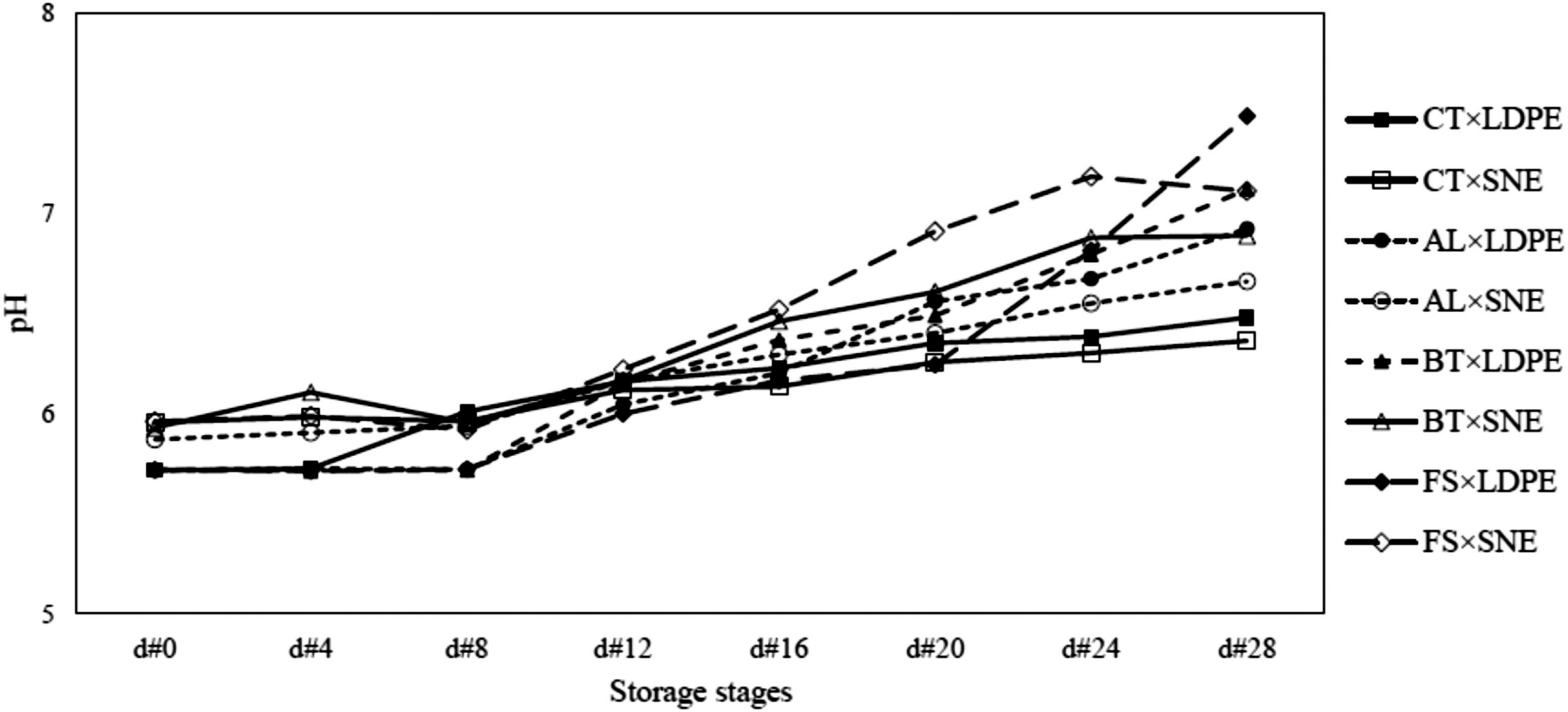
pH values related to the treatment combinations of FI × MP during the storage period.

The pH of garlic samples increased over the storage period (Figure 4). Fungal infection accelerated this process, while storage of cloves in SNE packaging had the opposite effect. Fungal pathogens are acidic, the presence of these agents causes the cloves to be water‐soaked and eventually their growth increases the pH of the crop (Booth & Kroll, 1989; Gsaller et al., 2018). The ANOVA showed that all effects of the studied treatments were significant on the pH changes (Table 1). The comparison of the means demonstrated that in infected samples pH increased more sharply than in CT ones. Case in point, the FS, BT, and AL pathogens had the greatest effect on pH increase, respectively. Unexpectedly, the use of SNE packaging accelerated the pH increase, while its effectiveness is much less in the presence of fungal infection. Moreover, the comparison of the means revealed that without considering d#0, the lowest and highest value of pH was seen in the treatment combinations of “CT × LDPE × d#4” (5.71) and “FS × LDPE × d#28” (7.48), respectively.

#### 3.2.2 TSS

**FIGURE 5:**
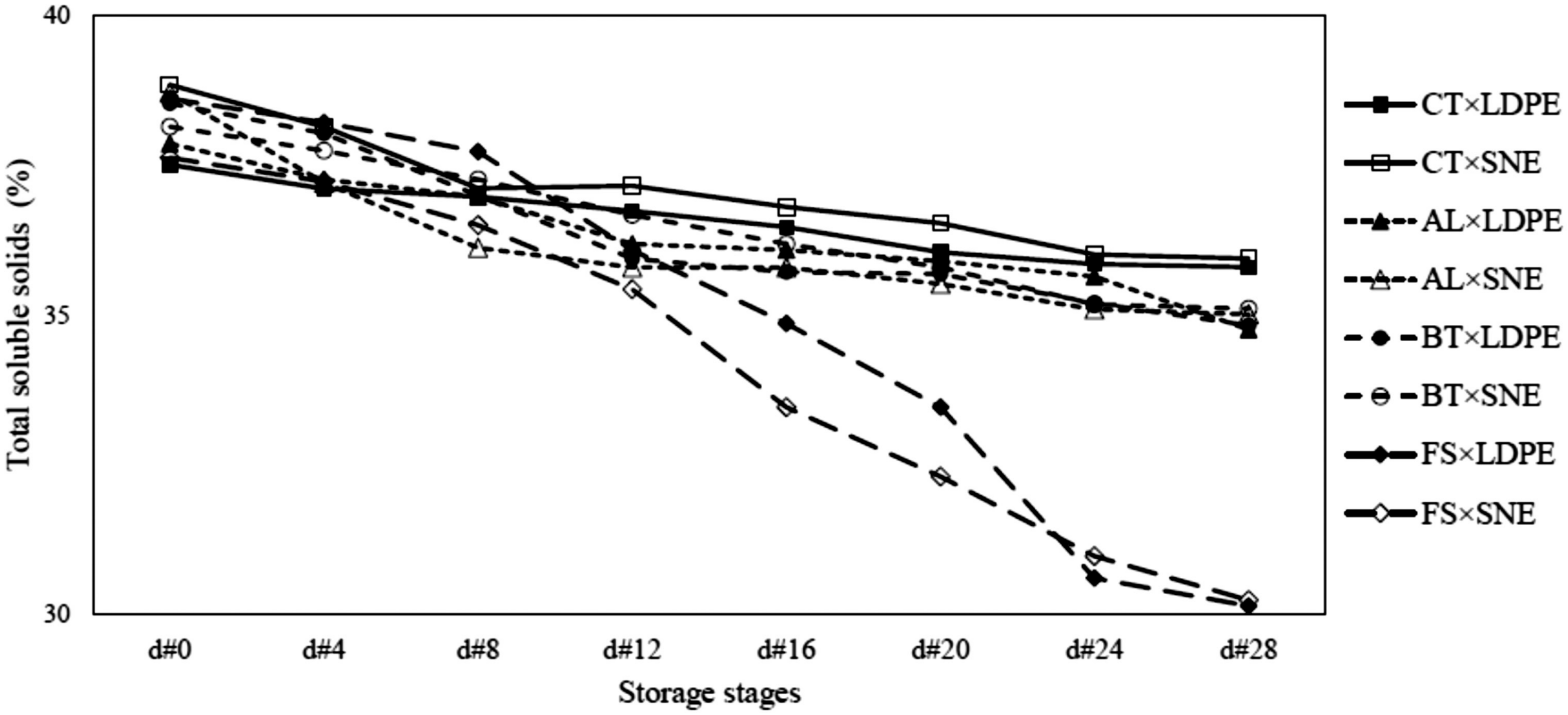
TSS values related to the FI × MP treatment combinations during the storage period.

The results exposed that the TSS decreased over the storage period. Fungal infection also increased the rate of TSS reduction in each storage stage. The TSS related to CT samples compared with other levels of FI treatment adopted the highest value. Notably, among the infected samples, the highest TSS in each specific storage stage belonged to the infection of BT, AL, and FS pathogens, respectively (Figure 5). In the presence of fungal infection during storage, more tissue destruction took place. Simultaneously, the cloves became water‐soaked and the TSS decreased. Moreover, the utilization of SNE packaging also reduced the rate of TSS reduction due to maintaining the quality of the clove's texture.

The ANOVA disclosed that all effects of studied treatments on the TSS changes were significant (Table 1). By comparing the means, it was found that the FS, AL, and BT pathogens had the greatest effect on TSS alteration, respectively. The lowest and highest TSS belonged to the treatment combinations of “FS × LDPE × d#28” and CT × SNE × d#0, respectively. Valverde‐Miranda et al. (2021), pointed out that during the storage period of cucumber, TSS reduction occurred increasingly and this feature can be used as an indicator for estimating the shelf‐life. Madhu et al. (2021) also noted that the effect of packaging materials as well as storage conditions had a significant influence on the TSS trait which is a qualitative index in the shelf‐life of garlic.

### 3.3 Changes in thermal properties

**FIGURE 6:**
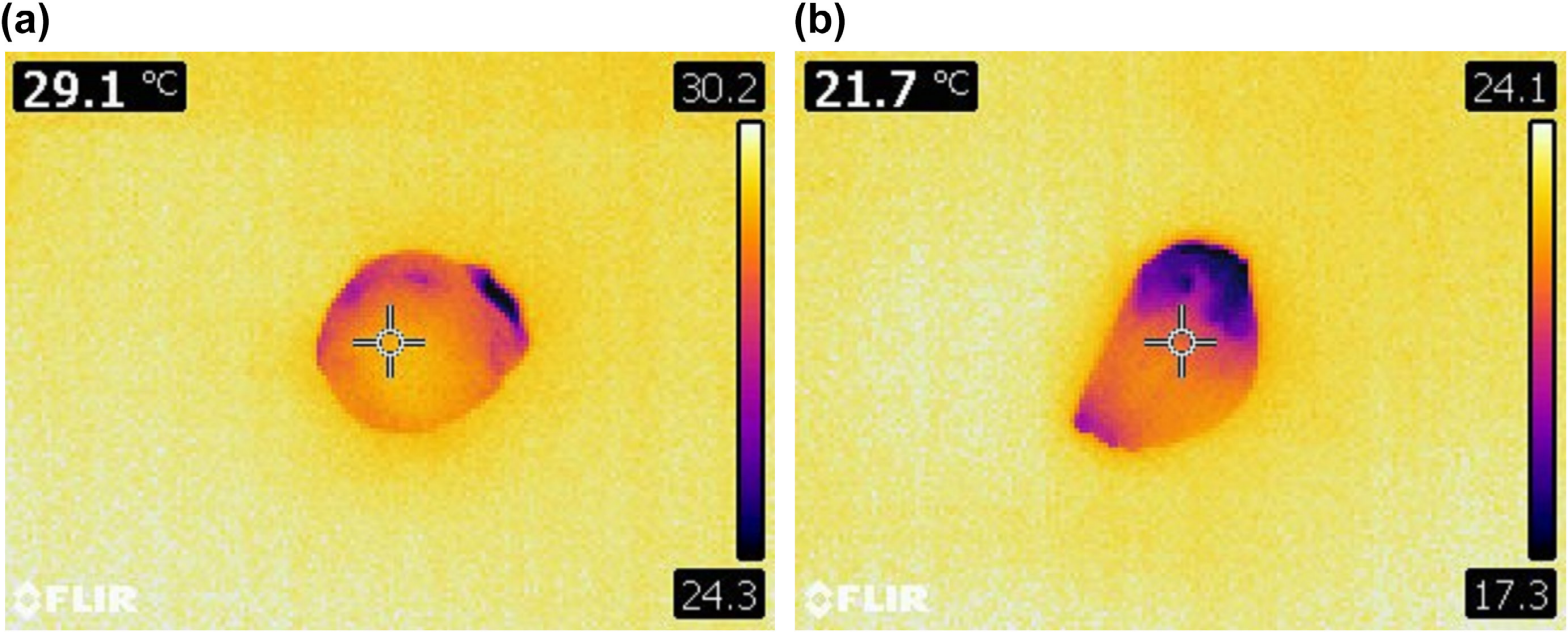
Typical thermal images of FS‐infected samples stored in the SNE packaging: (a) d#0, (b) d#28.

**FIGURE 7:**
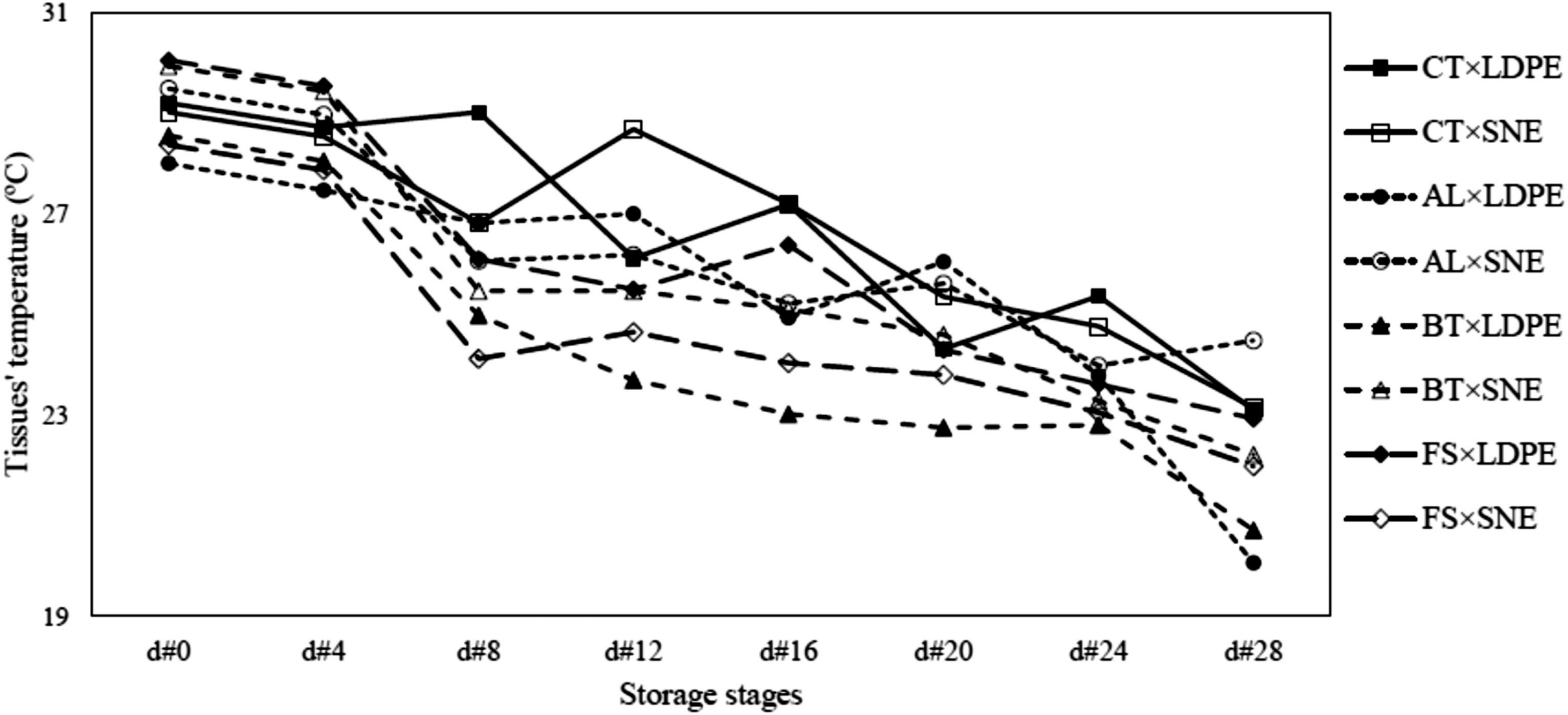
TT values related to the treatment combinations of FI × MP during the storage period.

A typical thermal image of garlic is shown in Figure 6. Thermal imaging of garlic surfaces demonstrated that the TT decreased over storage stages. Also, the fungal infection caused more falling in TT due to the more severe tissue destruction. Although the trend of TT changes was not regular in CT samples, this index decreased during the storage period. In contrast, the TT reduction rate associated with infected samples increased much more rapidly and systematically from d#8. As mentioned earlier, the use of SNE packaging due to high inhibitory properties (compared to LDPE) and its acceptable efficiency in the prohibition of tissue damage resulted in a reduced rate of the TT (Figure 7).

The ANOVA results exposed that the effects of FI and SD treatments on TT alteration were significant, while the effect of MP treatment was not meaningful. Moreover, the triple interaction effect of studied treatments on the TT alteration was significant at the level of 5%.

The comparison of the means also revealed that the levels of FS and BT infection had the greatest effect on TT reduction, respectively. Moreover, the comparison of the means clarified that the TT of samples on D#0 was almost equal, while the lowest TT belonged to the treatment combination of “AL × LDPE × d#28”. Paulsen et al. (2018), stated that the temperature of the product was strongly affected by respiration as well as the structure of its tissue. Hence, the use of packaging with high inhibitory properties could control the destructive factors and improve the shelf‐life of agricultural products.

### 3.4 Changes in mechanical properties

**FIGURE 8:**
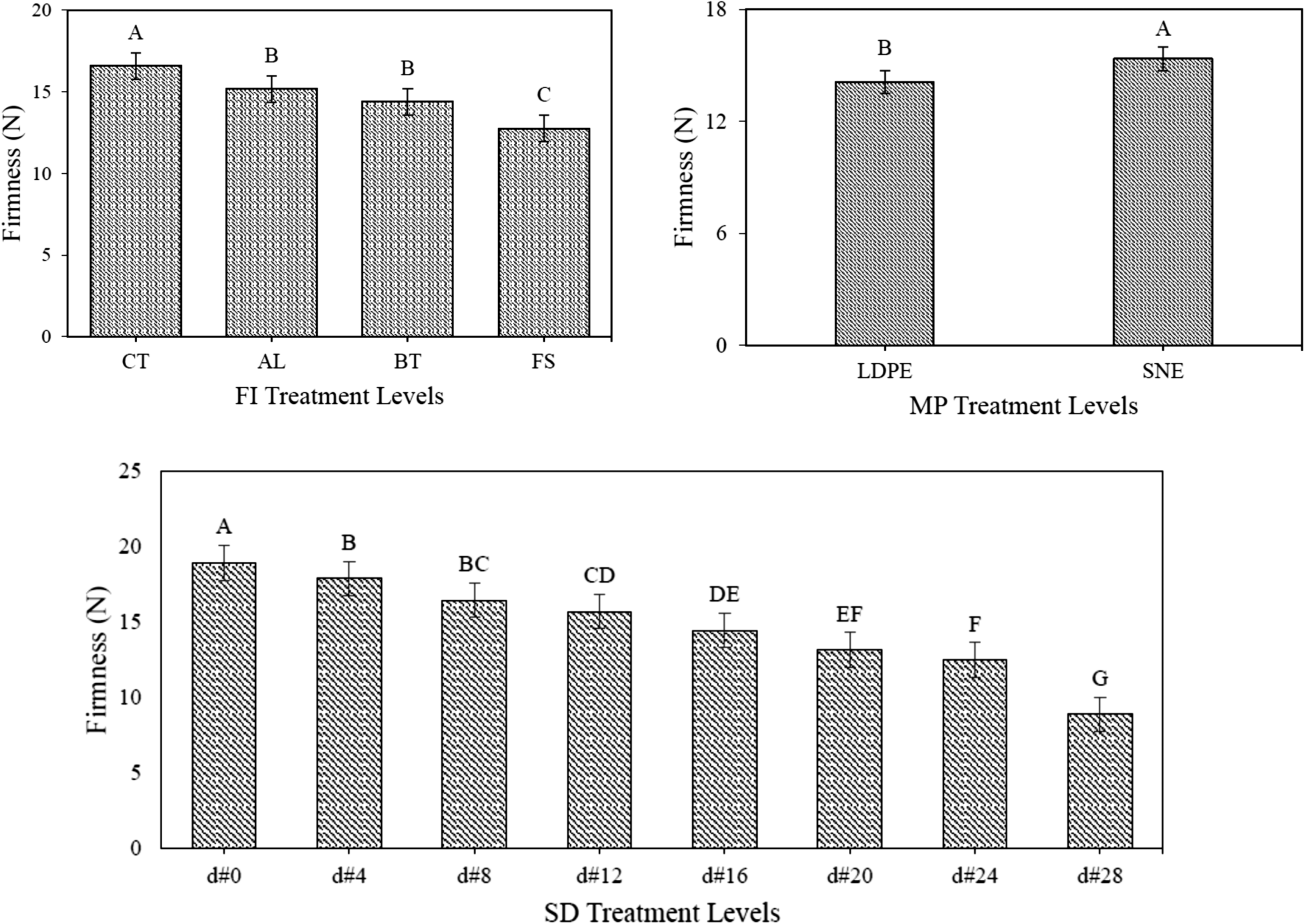
Comparison of the means of *F*_max_ values associated with each of the different levels of the studied treatments.

**FIGURE 9:**
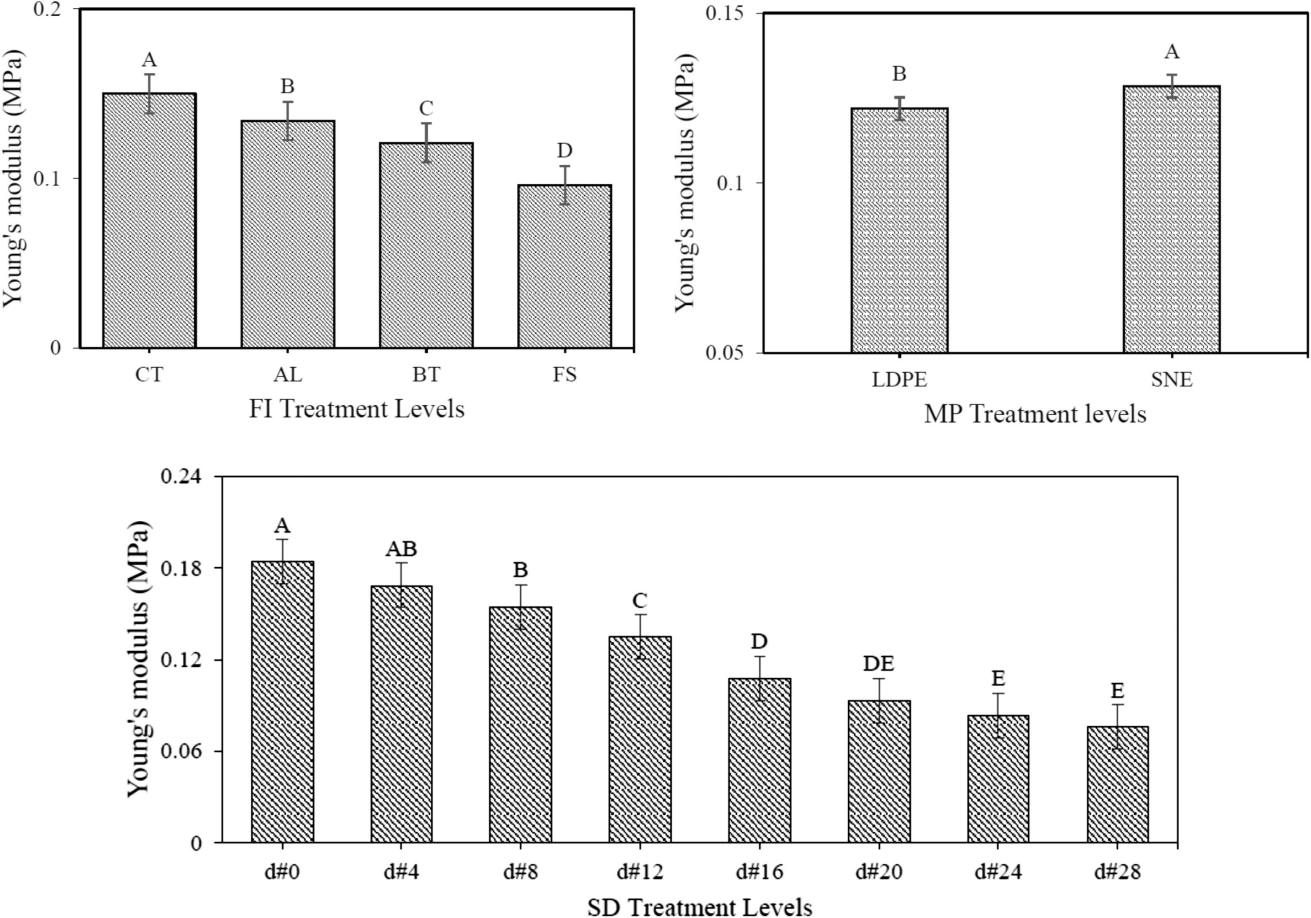
Comparison of the means of *E*_mod_ values associated with each of the different levels of the studied treatments.

Due to garlic respiration, the tissue structure lost its strength. Therefore, the *F*_max_ as well as *E*_mod_ decreased over the storage period. The fungal infection terminated to more tissue damage. Hence, the strength of infected garlic decreased more sharply than that of CT ones. Compared with LDPE, the use of SNE packaging maintained the quality of the samples in better conditions. Hence, the *F*_max_ as well as *E*_mod_ of the samples stored in the SNE decreased with a lower intensity (Figures 8 and 9).

The ANOVA consequence that only the simple effects of the studied treatment on mechanical traits were significant (Table 1). The comparison of the means exposed that the FI treatment had the greatest effect on tissue destruction and ultimately *F*_max_ as well as *E*_mod_ reduction, so that the lowest and highest mechanical traits were related to the CT‐ and FS‐infected samples, respectively. Moreover, there was no significant difference between the levels of infections to the AL and BT pathogens.

Lee et al. (2019), used nano‐composite films based on silver biopolymers to preserve foods. Following the results presented in this study, they stated that the presence of pathogens (fungal or microbial), can affect the mechanical properties of food which are considered as an important indicator in assessing the shelf‐life. They also pointed out that the use of new materials in food packaging can delay the destructive effects of pathogens.

### 3.5 Aroma variation during the storage

#### 3.5.1 PCA results

**FIGURE 10:**
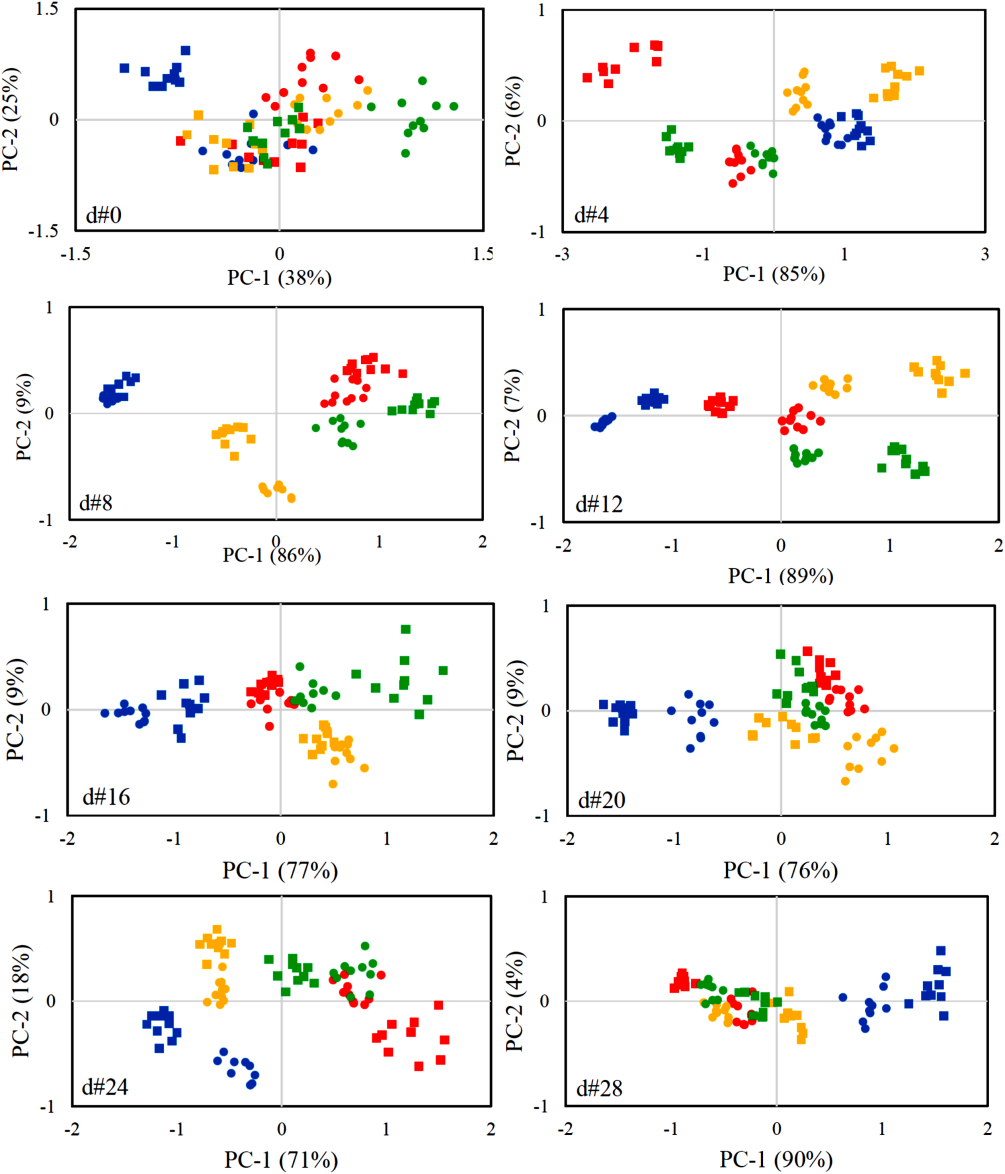
PCA results for the data obtained from the E‐Nose related to different treatment combinations of FI × MP; 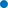 CT × LDPE, ■ CT × SNE, 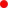 AL × LDPE, ■ AL × SNE, 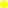 BT × LDPE, ■ BT × SNE, 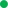 FS × LDPE, ■ FS × SNE.

According to the response changes in the array, clear results about the effect of the studied treatments on the aroma profile of garlic were not provided. Therefore, the utilization of the PCA was crucial. PCA outcomes revealed that immediately after inoculation (d#0), the PC‐1 and PC‐2 comprised 38% and 25% of the data, respectively (Figure 10). The score values showed that the pattern associated with different levels of FI and MP treatments could not be distinguished and the overlap was observed between all combinations. These results were predictable because the effects of the FI and MP treatments had not been enough to alter the aroma.

The PCA results on d#4 showed that the PC‐1 and PC‐2 included 85% and 6% of the inputs, respectively. The score patterns associated with the aroma profile of CT‐ and BT‐infected samples were located separately on the right axis of the PC‐1, while the scores of AL‐ and FS‐infected samples were overlapped on the left. In other words, it was possible to categorize the response of the array based on the presence of infection, but the type of infection could not take into account separate patterns. It is noteworthy that on d#4, it was not possible to categorize the score values based on MP treatment. Moreover, the MQ9, MQ6, MQ4, and MQ135 sensors (in the order of appearance) had the highest loading values in the patterning of scores on d#4.

The PCA results related to d#8, were more accurate. The PC‐1 and PC‐2 covered 86% and 9% of the input variables, respectively. The score patterns showed that the E‐Nose, in addition to the presence of infection, fully categorized its type. Besides, the E‐Nose was able to detect the different levels of MP treatment on d#8. Precisely, except for the samples infected with FS and BT pathogens, it was not possible to categorize the patterns based on the packaging type in the rest of the FI treatment levels. This point indicated that the garlic's aroma was more influenced by the FI and the MP treatment just controlled the destructive effects of pathogenic infection. Noteworthy, the sensors MQ9, MQ5, MQ135, MQ6, and MQ2 had the highest loading values on the patterning of scores in d#8, respectively.

It was found that up to d#12 and with the progression of pathogen infection, the effect of FI treatment on aroma profiles was more than the MP treatment. From d#16 onwards, there were overlaps between the profiles associated with AL‐ and FS‐infected samples. Subsequently, after d#20, the patterns of scores could not be categorized according to the type of infection, and only the profile of CT samples discriminated.

Buratti et al. (2018) investigate the applicability of a commercial E‐Nose (PEN2) for the characterization of edible olive oils. The PCA accuracy was 72.8%–76.5%. Their results exposed a good discrimination between different quality levels. They revealed that the storage of agricultural products causes aroma alteration which can be controlled by novel solutions such as packaging. Wilson (2018), stated that the E‐Nose can be extended as a fast, accurate, and non‐destructive solution for diverse issues such as health checking and diagnostic applications.

#### 3.5.2 LDA results

According to PCA results, it was found that different levels of the studied treatments affect the aroma of garlic as well as the response of the sensor array. In the next step, there was an urge to implement the classification approaches to determine how the aroma patterns could be grouped if the classifiers were trained well. The dataset applied for the LDA was the identical one adopted for PCA. In class membership, method defaults were used for prior probabilities. The LDA results exposed that the accuracy of classification based on the FI treatment had an ascending‐descending trend (Table 2). The optimal classification was obtained at d#12 with an accuracy of 100%. Expectedly, the FI treatment still did not influence on the aroma profiles of the samples on d#0. Therefore, the classification of patterns was not complete. The aroma profile classification of the samples was done more accurately on d#4. The CT and BT‐infected classes were completely classified but there was a misclassification between the AL‐ and FS‐infected classes. Similar to d#4, the similar trend continued in d#8, with the difference that the classification accuracy increased to 97.5%.

**TABLE 2:** Results of LDA and BPNN classification based on the FI and MP treatments.

Classification basis	Classifier name	Storage stage							
		d#0	d#4	d#8	d#12	d#16	d#20	d#24	D#28
Accuracy (%)									
FI levels	LDA	70	93.75	97.5	100	97.5	96.25	95	92.5
	BPNN	75	97.5	100	100	98.8	97.5	96.3	92.5
MP levels	LDA	75	78.75	83.75	85	86.25	87.5	88.75	93.75
	BPNN	73.8	83.8	87.5	88.8	88.8	98.8	100	100

On d#12, all the aroma profiles of the samples were classified into the correct classes. In contrast, from d#16 to d#28, the misclassification between AL‐ and FS‐infected classes increased. The progression of pathogenic infection resulted in more differentiation of aroma profiles related to the CT‐ and BT‐infected classes from the AL‐ and FS‐infected classes. The results of LDA and PCA approaches were consistent. It can be concluded that the existence of fungal infection and its type can be detected by examining the garlic's aroma, especially in the early stages of storage. This point can help in the early isolation of non‐contaminated samples from infected ones to prevent upcoming potential damages. Although the LDA was not able to fully classify garlic aroma based on the MP treatment, classification accuracy increased as the levels of MP treatment progressed (Table 2). This result revealed that the effect of MP treatment is evident in the long term to the extent that it overshadows the destructive impact of the FI treatment.

#### 3.5.3 BPNN results

Due to the complexity of the treatment's combinations matrix, 14 neurons were selected for the hidden layer of the BPNN algorithm by the trial‐and‐error approach. The array response was considered as the input vector, and the different levels of studied treatments were treated as the output vector. Hence, the network topology for the classification was designed 9‐14‐4 (based on the FI treatment) and 9‐14‐2 (based on the MP treatment). The utilized dataset was unmethodically distributed into three sets, that is, the training (70%), validation (15%), and test (15%) sets.

Same as the LDA, the accuracy of BPNN classification based on the FI treatment followed an ascending‐descending trend (Table 2). The BPNN classified completely the levels of FI treatment from d#8 to d#12. The accuracy of BPNN classification based on the FI treatment decreased from d#16. This reduction continued until the end of the storage period. The BPNN utilization disclosed that as the pathogenic infection progressed, the aroma of garlic also altered throughout the storage period.

In the classification of aroma profiles of garlic based on the MP treatment by the BPNN method, the accuracy increased over time (Table 2). Although the LDA could not fully classify the aroma profile of the samples in any of the storage stages, the BPNN classified fully correct from d#24 onwards. As mentioned before, the presence of fungal infection caused the destruction of the garlic over time. After a certain point in time, the aroma of garlic no longer changes. These results proved that different levels of MP treatment delayed this incident. Therefore, the effect of MP treatment on the categorization of garlic aroma was greatest in d#28. In general, comparing the LDA and BPNN outcomes disclosed that the results of the BPNN were better than the LDA. Besides, the BPNN results demonstrated the effect of the studied treatments more clearly.

Numerous researchers have used various classifiers to inspect the pattern categorization of samples during storage. Karami et al. (2020), assessed the shelf‐life of edible oil using E‐Nose in a 150‐day storage period. Data analysis was also achieved by diverse approaches including LDA, quadratic discriminant analysis (QDA), and support vector machine (SVM). According to the results, the classification accuracies of LDA, QDA, and SVM were 94.4%, 95.8%, and 96.25%, respectively. Güney and Atasoy (2020), tested the freshness of fish by utilizing of commercial E‐Nose composed of eight different MOS sensors. To enhance accuracy in determining the freshness, the k‐Nearest Neighbors, SVM, and LDA approaches were employed. They stated that the presence of any factor that disturbs the balance of the product during the shelf‐life can affect the aroma of the product and ultimately overshadow the performance of classification methods.

### 3.6 PLS results

In the PLS approach, the response of the sensor array and the evaluated traits were used as the input and output of the model, respectively. The regression model related to the evaluated traits is also presented in Equation 3. The *Y*_Characteristic_ expresses the predicted values. In addition, a constant coefficient of a_0_ and variable coefficients of a_1_–a_9_ represent the predictor variables (response of the MOS sensors). In this regard, Table 3 shows the regression coefficients of the models related to each of the evaluated traits.

(3) $$\begin{array}{cc} Y_{\text{Characteristic}}= & \;a_{0}+a_{1}\;\left(\text{MQ2}\right)+a_{2}\;\left(\text{MQ3}\right)+a_{3}\;\left(\text{MQ4}\right)+a_{4}\;\left(\text{MQ5}\right)+a_{5}\;\left(\text{MQ6}\right)\\ & \;+a_{6}\;\left(\text{MQ7}\right)+a_{7}\;\left(\text{MQ8}\right)+a_{8}\;\left(\text{MQ9}\right)+a_{9}\;\left(Q135\right) \end{array}$$

**TABLE 3:** Regression coefficients estimated by the model obtained from the PLS approach.

Trait	a_0_	a_1_	a_2_	a_3_	a_4_	a_5_	a_6_	a_7_	a_8_	a_9_	*R* ^2^
WL	9.95	−6.50	−1.26	−4.99	5.28	2.14	−5.87	−28.55	30.48	−0.36	.83
CC	10.95	11.14	−11.37	−3.12	5.15	4.11	−2.41	−9.19	−3.64	−7.32	.87
TT	23.05	0.87	0.62	−1.09	2.22	0.33	0.58	38.82	−1.67	−0.91	.84
pH	7.11	0.36	−0.34	−0.05	−0.72	−0.04	0.66	−0.03	−0.01	0.04	.75
TSS	35.95	−3.71	2.14	0.06	1.15	−1.04	−0.29	1.68	1.02	1.26	.70
*F*_max_	6.32	7.75	−7.82	9.90	−0.16	−16.21	9.26	15.51	1.88	3.06	.32
*E*_mod_	2.70	−0.10	−0.08	−0.21	−0.14	−0.15	−0.10	−0.01	−0.03	−0.14	.32

Abbreviations: CC, color changes; *E*_mod_, Young's modulus; *F*_max_, firmness; TSS, total soluble solids; TT, tissue temperature; WL, weight loss.

In the case of physical traits, the performance of WL modeling was satisfactory and a high correlation was observed. 83% of the E‐Nose responses were correlated to the WL. In predicting the WL, the MQ9, MQ135, and MQ5 sensors had the highest loading value in determining the correlation, respectively. Also, the CC modeling based on aroma profiles exposed a good performance (*R* ^2^ = .87). The MQ5, MQ6, and MQ9 sensors had the highest loading values in the interpretation of the CC trend, respectively. Moreover, the results of TT modeling showed that 84% of its changes were correlated to the response changes of the array. The MQ9, MQ135, and MQ6 sensors had the highest loading values in the correlation between TT alteration and aroma profile patterning.

In the case of chemical traits, the performance of the PLS model demonstrated that about 75% of response changes of the array were equated to pH alteration. During storage, changes in pH had the highest correlation with changes in the response of MQ4, MQ7, and MQ2 sensors, in the order of appearance. By modeling the correlation between changes in TSS and sensor array response, about 70% of the response changes of the array were touched by the TSS alteration. In remarking the TSS alteration, the MQ6, MQ5, and MQ9 sensors had the highest loading values, respectively.

In the case of mechanical traits, the performance of the PLS in modeling the *F*_max_ during the storage was 32%. The MQ135, MQ4, and MQ6 sensors had the highest loading values in determining the correlation between the changes in the *F*_max_ and the response of the sensor array. The PLS results in predicting the *E*_mod_ of garlic during storage released that about 32% of the dependent variable changes were influenced by independent variable changes. The trend of changes in the *E*_mod_ had the highest correlation with MQ4, MQ135, and MQ9 sensors, respectively.

## 4 CONCLUSIONS

Garlic was stored for 28 days to evaluate the effect of FI, MP, and SD treatments on the dominant qualitative traits in the storage. The effect of the MP treatment increased over time and gradually overshadowed the destructive effects of the FI treatment. During the storage period, the values associated with WL, CC, and pH increased, while the values of TSS, *F*_max_, and *E*_mod_ decreased. The presence of fungal infection intensified the trend, while the use of packaging with Nanomaterial slowed down the destructive incident. The E‐Nose had acceptable sensitivity to the aromas related to different treatment combinations. The PCA fully categorized the FI and MP treatments from d#8 and d#12. The PCA results disclosed that the garlic's aroma was more influenced by the FI than the MP treatment. The best performances of classifiers were obtained from d#8 to d#12. The effects of studied treatments were detectable in the early stages of storage by the E‐Nose. The PLS revealed that the aroma alteration had a high correlation with the variations of studied traits (*R* ^2^ ≥ 0.7), apart from the mechanical properties. Therefore, the E‐Nose technique can be an alternative to destructive approaches for the assessment of the qualitative traits in the storage.

## AUTHOR CONTRIBUTIONS

**Alireza Makarichian:** Data curation (equal); methodology (equal); validation (equal); writing – original draft (equal). **Ebrahim Ahmadi:** Investigation (equal); supervision (equal); validation (equal); writing – review and editing (equal). **Reza Amiri Chayjan:** Investigation (equal); resources (equal); supervision (equal). **D. M. Zafari:** Investigation (equal); methodology (equal); validation (equal).

## ACKNOwledgements

Bu‐Ali Sina University is appreciated for supporting this research in terms of supplying laboratory materials and equipment.

## Data Availability

The data that support the findings of this study are available from the corresponding author upon reasonable request.
